# Senescence and senotherapies in biliary atresia and biliary cirrhosis

**DOI:** 10.18632/aging.204700

**Published:** 2023-05-18

**Authors:** Giulia Jannone, Eliano Bonaccorsi Riani, Catherine de Magnée, Roberto Tambucci, Jonathan Evraerts, Joachim Ravau, Pamela Baldin, Caroline Bouzin, Axelle Loriot, Laurent Gatto, Anabelle Decottignies, Mustapha Najimi, Etienne Marc Sokal

**Affiliations:** 1 Pediatric Hepatology and Cell Therapy Unit, Institut de Recherche Expérimentale et Clinique, UCLouvain, Brussels, Belgium; 2 Abdominal Transplantation Unit, Department of Surgery, Cliniques Universitaires Saint-Luc, UCLouvain, Brussels, Belgium; 3 Pediatric Surgery and Transplantation Unit, Department of Surgery, Cliniques Universitaires Saint-Luc, UCLouvain, Brussels, Belgium; 4 Department of Anatomopathology, Cliniques Universitaires Saint-Luc, UCLouvain, Brussels, Belgium; 5 IREC Imaging Platform (2IP), Institut de Recherche Expérimentale et Clinique, UCLouvain, Brussels, Belgium; 6 Computational Biology and Bioinformatics Unit, de Duve Institute, UCLouvain, Brussels, Belgium; 7 Genetic and Epigenetic Alterations of Genomes Group, de Duve Institute, UCLouvain, Brussels, Belgium

**Keywords:** senescence, senotherapy, liver, biliary cirrhosis, biliary atresia

## Abstract

Background: Premature senescence occurs in adult hepatobiliary diseases and worsens the prognosis through deleterious liver remodeling and hepatic dysfunction. Senescence might also arises in biliary atresia (BA), the first cause of pediatric liver transplantation. Since alternatives to transplantation are needed, our aim was to investigate premature senescence in BA and to assess senotherapies in a preclinical model of biliary cirrhosis.

Methods: BA liver tissues were prospectively obtained at hepatoportoenterostomy (n=5) and liver transplantation (n=30) and compared to controls (n=10). Senescence was investigated through spatial whole transcriptome analysis, SA-β-gal activity, p16 and p21 expression, γ-H2AX and senescence-associated secretory phenotype (SASP). Human allogenic liver-derived progenitor cells (HALPC) or dasatinib and quercetin (D+Q) were administrated to two-month-old Wistar rats after bile duct ligation (BDL).

Results: Advanced premature senescence was evidenced in BA livers from early stage and continued to progress until liver transplantation. Senescence and SASP were predominant in cholangiocytes, but also present in surrounding hepatocytes. HALPC but not D+Q reduced the early marker of senescence p21 in BDL rats and improved biliary injury (serum γGT and *Sox9* expression) and hepatocytes mass loss (*Hnf4a*).

Conclusions: BA livers displayed advanced cellular senescence at diagnosis that continued to progress until liver transplantation. HALPC reduced early senescence and improved liver disease in a preclinical model of BA, providing encouraging preliminary results regarding the use of senotherapies in pediatric biliary cirrhosis.

## INTRODUCTION

Senescence is a process of cellular ageing, resulting in metabolic modifications and in an irreversible cell cycle arrest [1]. Various cellular changes reflect this complex phenomenon, including upregulation of cell cycle inhibitors (e.g. p21 and p16) and anti-apoptotic pathways, increased lysosomal protein content and senescence-associated beta-galactosidase (SA-β-gal) activity, accumulation of DNA damage foci (e.g. γ-H2AX and/or 53BP1-positive foci), and a senescence-associated secretory phenotype (SASP). Because there are no completely specific senescence features, the gold standard is to assess cellular senescence through the evaluation of multiple markers [2].

Accelerated senescence occurs in numerous chronic adult hepatobiliary diseases and has been associated to disease severity and cirrhosis [3–5]. The initial biliary damage in cholangiopathies induces premature senescence in cholangiocytes, and senescence subsequently spreads due to autocrine and paracrine transmission through the SASP [6–8]. Senescence progression worsens the disease prognosis as the SASP directly participates in the deleterious tissue remodeling and leads to liver dysfunction by inducing senescence in surrounding hepatocytes [9]. Furthermore, clearance of senescent cells or inhibition of the paracrine transmission of senescence improves liver function, histological fibrosis or steatosis in mouse models of hepatobiliary diseases [9–13]. Existing data demonstrate that liver disease improves when senescence decreases, thus supporting the development of senotherapeutics that could be translated to clinical applications.

Biliary atresia (BA) is a severe pediatric disease caused by the progressive fibro-inflammatory obliteration of extrahepatic bile ducts, leading to biliary cirrhosis and end-stage liver disease [14]. Despite the fact that BA is the first cause of liver transplantation in children, its underlying mechanisms are still not completely elucidated [14, 15]. A hepatoportoenterostomy procedure can be attempted in case of early diagnosis, but liver transplantation remains the only curative treatment in about two thirds of the cases [16]. A few studies described premature senescence in BA by using single technical approaches, but the possibility of using anti-senescence therapies was never explored in pediatric biliary cirrhosis [17–20].

In brief, premature senescence worsens the prognosis of adult chronic hepatobiliary diseases and some evidence suggests that this phenomenon could also occur in BA, which is the leading cause of liver transplantation in children. As there is a need for new therapies to avoid or delay liver transplantation in pediatric biliary cirrhosis, the aim of our work was to investigate premature senescence in BA through a multi-technical approach and to assess senotherapies in a preclinical model of biliary cirrhosis.

## RESULTS

### Premature senescence is present at diagnosis in BA and progresses until liver transplantation

Liver tissue samples were obtained from thirty BA patients undergoing liver transplantation (BA late group) and five patients during hepatoportoenterostomy procedure (BA early group), the latter procedure being performed very shortly after the diagnosis. All BA patients were pediatric, while the ten control livers were obtained from pediatric and adult individuals (four adults aged 28 – 41 years old). General characteristics of the study population are summarized in Supplementary Table 1. As expected, fibrosis and biliary proliferation increased in BA livers as COL1A1 gene expression was already elevated in early stage BA, while histological fibrosis and CK19 gene expression increased in late stage BA (Supplementary Figure 1).

**Figure 1:**
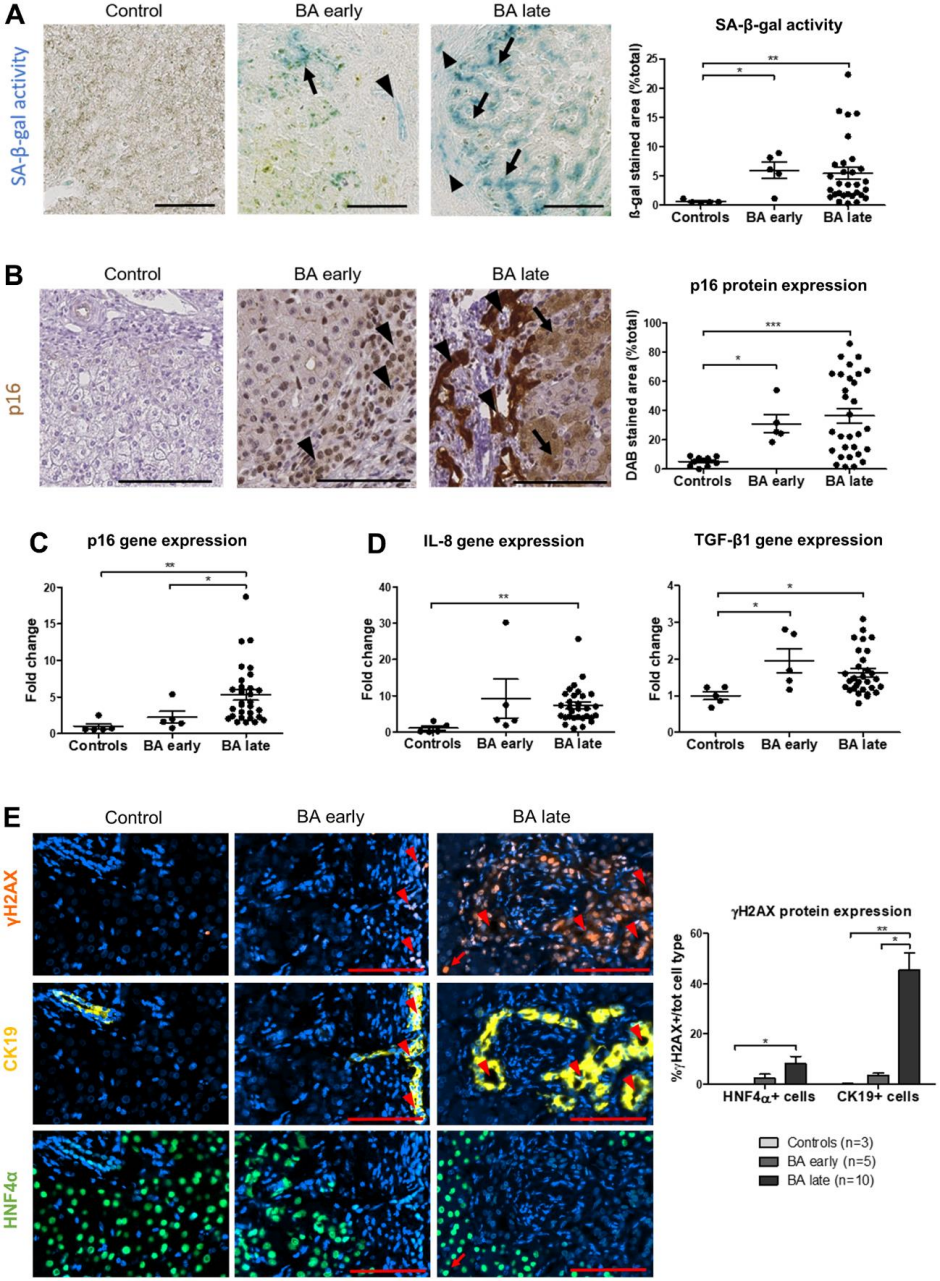
**Senescence increases in BA livers and predominates in cholangiocytes and perinodular hepatocytes.** (**A**) SA-β-gal activity increases in cholangiocytes (arrowheads) and surrounding perinodular hepatocytes (arrows) in BA livers. (**B**) p16 protein expression also increases in cholangiocytes (arrowheads) and surrounding perinodular hepatocytes (arrows) in BA livers. (**C**) Gene expression of p16 progresses until liver transplantation in BA. (**D**) Gene expression of SASP markers IL-8 and TGF-β1 increase in BA livers versus controls. (**E**) DNA damage γH2AX-positive foci increase in both hepatocytes and cholangiocytes in BA livers and progress until liver transplantation in cholangiocytes. BA: biliary atresia; DAB: 3,3′-diaminobenzidine; SA-β-gal: senescence-associated beta-galactosidase; Data is presented as mean ± SEM; *p≤0.05, **p<0.01, ***p<0.001. Scale bars = 100 μm.

**Figure 2:**
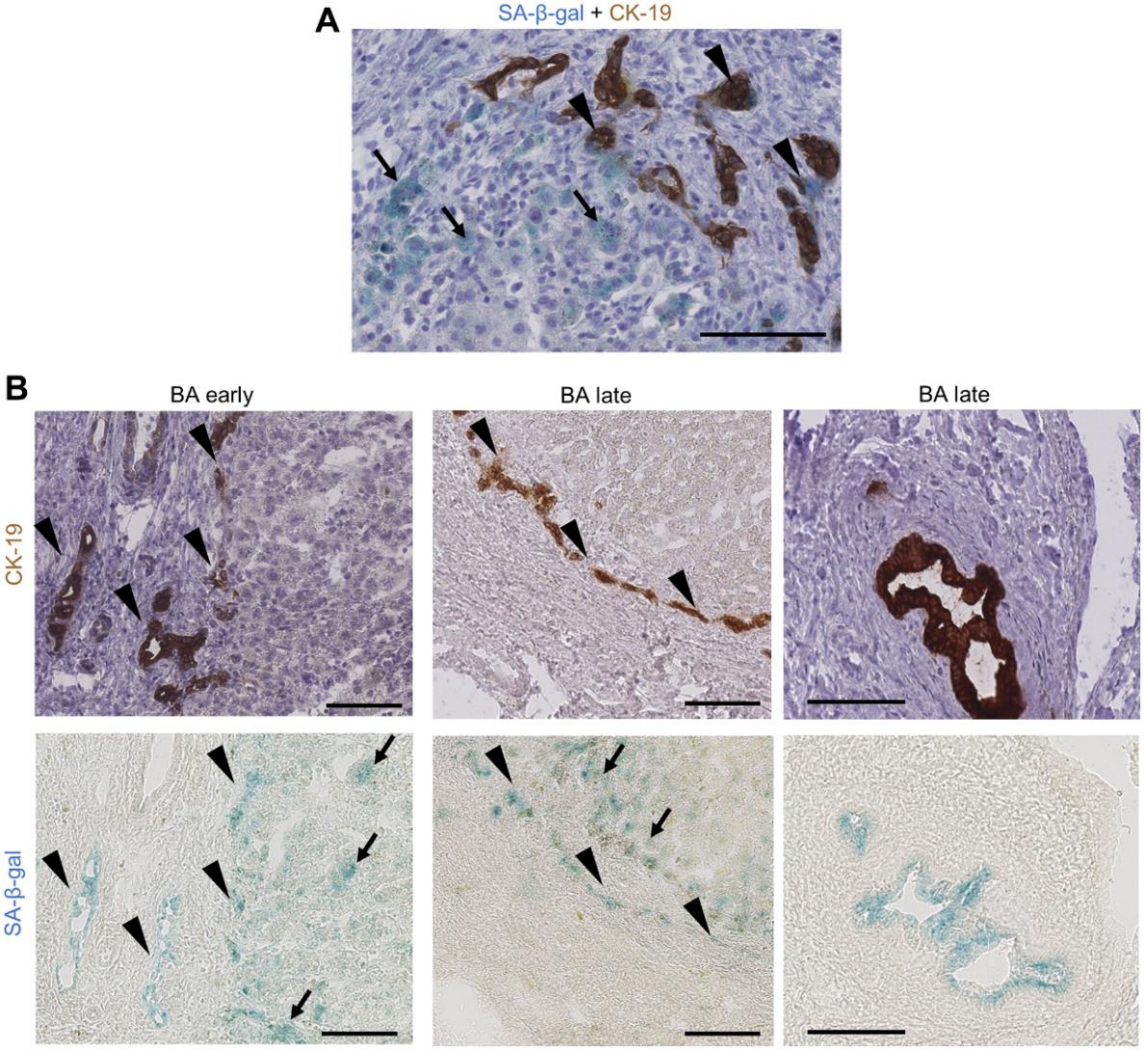
**Cholangiocytes and perinodular hepatocytes display cellular senescence in BA livers.** (**A**) Co-staining of SA-β-gal activity and CK-19 IHC on BA livers: some bile ductules cholangiocytes show staining co-localization (arrowheads) while perinodular hepatocytes are only positive for SA-β-gal (arrows); (**B**) Serial staining of SA-β-gal activity and CK-19 IHC on BA livers cryosections. Left and middle images: bile ductules (arrowheads) and perinodular hepatocytes (arrows) are positive for SA-β-gal in both early and late stage BA. Right images: remaining large septal bile duct display SA-β-gal activity. BA: biliary atresia; IHC: immunohistochemistry; SA-β-gal: senescence-associated beta-galactosidase. Scale bars = 100 μm.

Senescence was evidenced in BA through multiple markers and techniques, including SA-β-gal activity, p16 protein and gene expression, γH2AX protein expression and SASP markers gene expression elevation (IL-8 and TGF-β1) (Figure 1A–1D and Supplementary Figure 2B). Of note, p16 and IL-8 gene expression significantly increased only in late stage BA (Figure 1C, 1D). Advanced premature senescence was already present in the BA early group, as suggested by an increased protein expression of p16 but not p21, p16 being described as a late marker of senescence (Figure 1B and Supplementary Figure 2A) [21]. However, there was still a clear progression of senescence between the time of diagnosis and liver transplantation (Figure 1C, 1E). Interestingly, senescence development was correlated to disease progression (Supplementary Figure 1B, 1C). Senescence was predominant in cholangiocytes and also present in surrounding perinodular hepatocytes (Figures 1A, 1B, 1E, 2). When we investigated the telomeric DNA damages in late stage BA, γH2AX-positive DNA damage foci were evenly distributed in the whole DNA sequence without any specific accumulation in telomeres (Supplementary Figure 2C). However, the absolute increase in the number of telomere damage foci might per se induce senescence in the affected cells [2].

Our results show that premature senescence was already advanced at diagnosis in BA and continued to progress until liver transplantation. Senescence was predominant in cholangiocytes and perinodular hepatocytes, corroborating the hypothesis of a paracrine transmission of senescence through elevated SASP markers.

### Digital spatial transcriptomic analysis in BA livers confirms that senescence and SASP predominate in cholangiocytes and progress with disease stage

**Figure 3:**
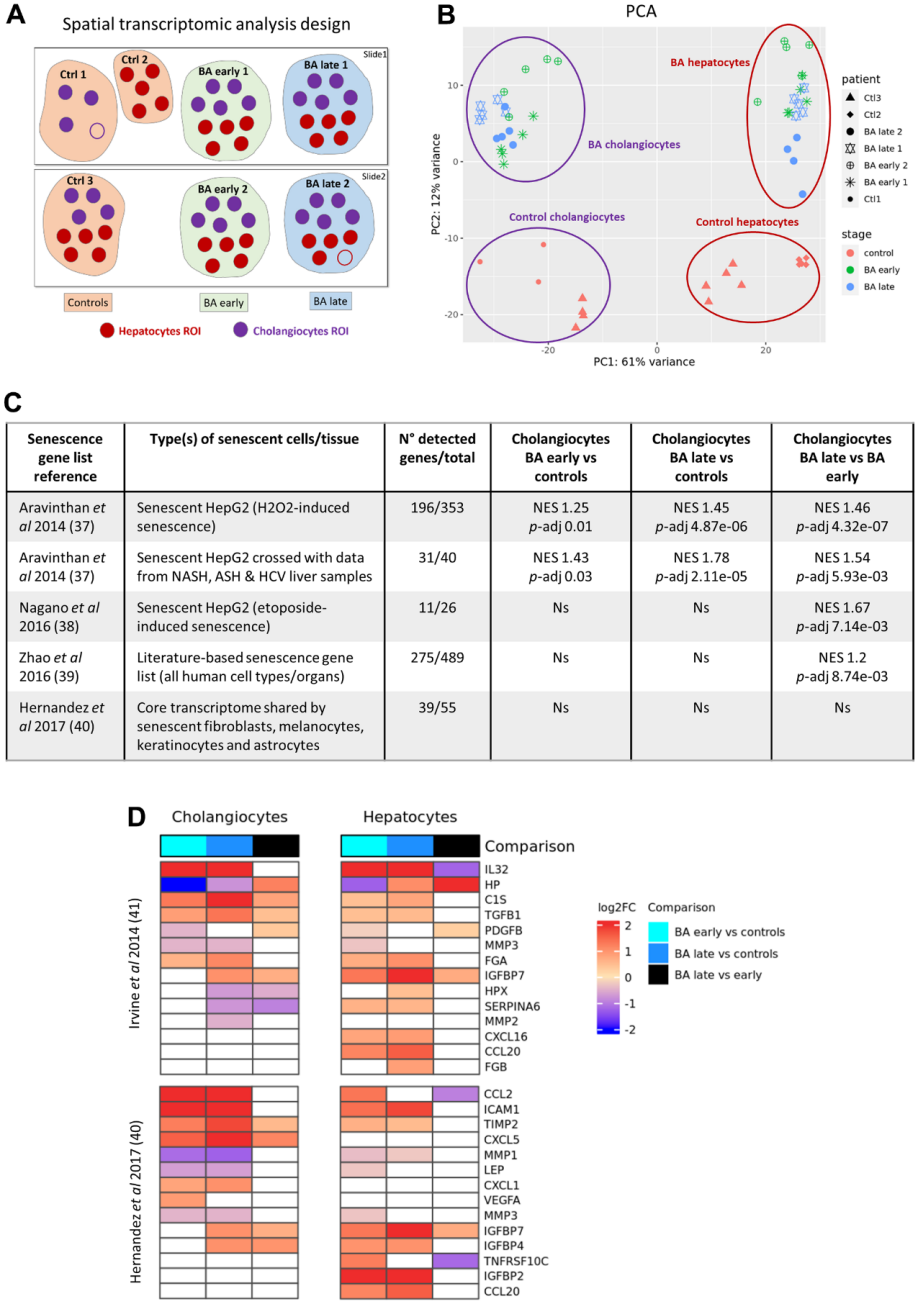
**Digital spatial whole transcriptomic analysis in BA livers.** (**A**) Design of the analysis. (**B**) PCA of the dataset. (**C**) Enrichment analysis of published senescence gene lists between cholangiocytes subgroups. (**D**) Heatmaps of SASP genes obtained from two different published datasets in cholangiocytes and hepatocytes subgroups. BA: biliary atresia; Ctrl: control; FC: fold change; NES: normalized enrichment score; *p*-adj: adjusted *p*-value; PCA: principal component analysis; ROI: region of interest; SASP: senescence-associated secretory phenotype.

To further assess the localization and progression of cellular senescence, we performed a digital spatial whole transcriptomic analysis on BA and control liver samples. Both cholangiocytes and hepatocytes regions of interest (ROI) were selected in BA early, BA late and control livers (Figure 3A). Principal component analysis (PCA) revealed that the transcriptomes differed according to cell type (cholangiocytes versus hepatocytes) and disease stage (Figure 3B). Early and late stage BA transcriptomes were clustered together and differed from controls for each cell type. Differential expression analysis was performed between disease stages subgroups for each cell type and data is available for the main differentially expressed genes in Supplementary Figure 3. Gene ontology (GO) enrichment analysis of biological processes (MSigDB Collection: C5 GO BP) and hallmark gene sets enrichment analysis (MSigDB Collection: H) were also performed on our dataset (Supplementary Figure 4). GO terms that appeared to be the most significant when comparing genes differentially expressed between diseased cholangiocytes versus controls corresponded to “supramolecular fiber organization”, “cell adhesion” and “ion transport” categories, in line with the disruption of apical-basal polarization described in BA cholangiocytes [22].

Enrichment analysis of senescence gene lists available in the literature was then performed in our dataset (Figure 3C). A geneset corresponding to genes that were upregulated in senescent HepG2 cells as well as in both senescent HepG2 and diseased human adult livers was significantly enriched in diseased cholangiocytes (BA early and late stages) as compared to controls, supporting the presence of senescence in BA cholangiocytes [23]. The same gene lists as well as two other datasets obtained from senescent HepG2 cells and from an organ-independent literature-based senescence signature database were significantly enriched in BA late stage cholangiocytes as compared to BA early stage, underlying the progression of senescence until liver transplantation in BA cholangiocytes [24, 25]. Volcano plots and detailed results are available in Supplementary Figure 5 for the leading edge genes of the enrichment analysis. In contrast, a core transcriptome database obtained from senescent human fibroblasts, melanocytes, keratinocytes and astrocytes was not significantly enriched in our diseased cholangiocytes, underlying that the transcriptome of senescent cells is highly heterogeneous depending on the senescent organ [26]. None of those publically available senescence datasets were enriched in our diseased hepatocytes. Various SASP genes obtained from two published datasets were differentially expressed in both diseased cholangiocytes and hepatocytes in our cohort (Figure 3D) [26, 27]. The two most overexpressed SASP factors in diseased cholangiocytes as compared to controls were IL-32 and CCL2 (log2 fold change > 2.5; *p*-adj < 1x10^-20^). IL-32 is a pro-inflammatory cytokine that was evidenced before in BA livers, while CCL2 has been associated to the paracrine transmission of senescence in cholangiopathy [28, 29]. A publically available SASP gene list obtained from senescent HepG2 cells was significantly enriched in BA late stage cholangiocytes as compared to BA early stage (NES 1.6; *p*-adj 0.007), and in diseased cholangiocytes as compared to hepatocytes (NES 1.47; *p*-adj 0.04) [27]. Altogether, those results confirm the progressive development of senescence and SASP in cholangiocytes until liver transplantation and highlight the predominance of senescence in BA cholangiocytes as compared to hepatocytes.

### Senescence progresses from cholangiocytes to hepatocytes after bile duct ligation surgery in rats

**Figure 4:**
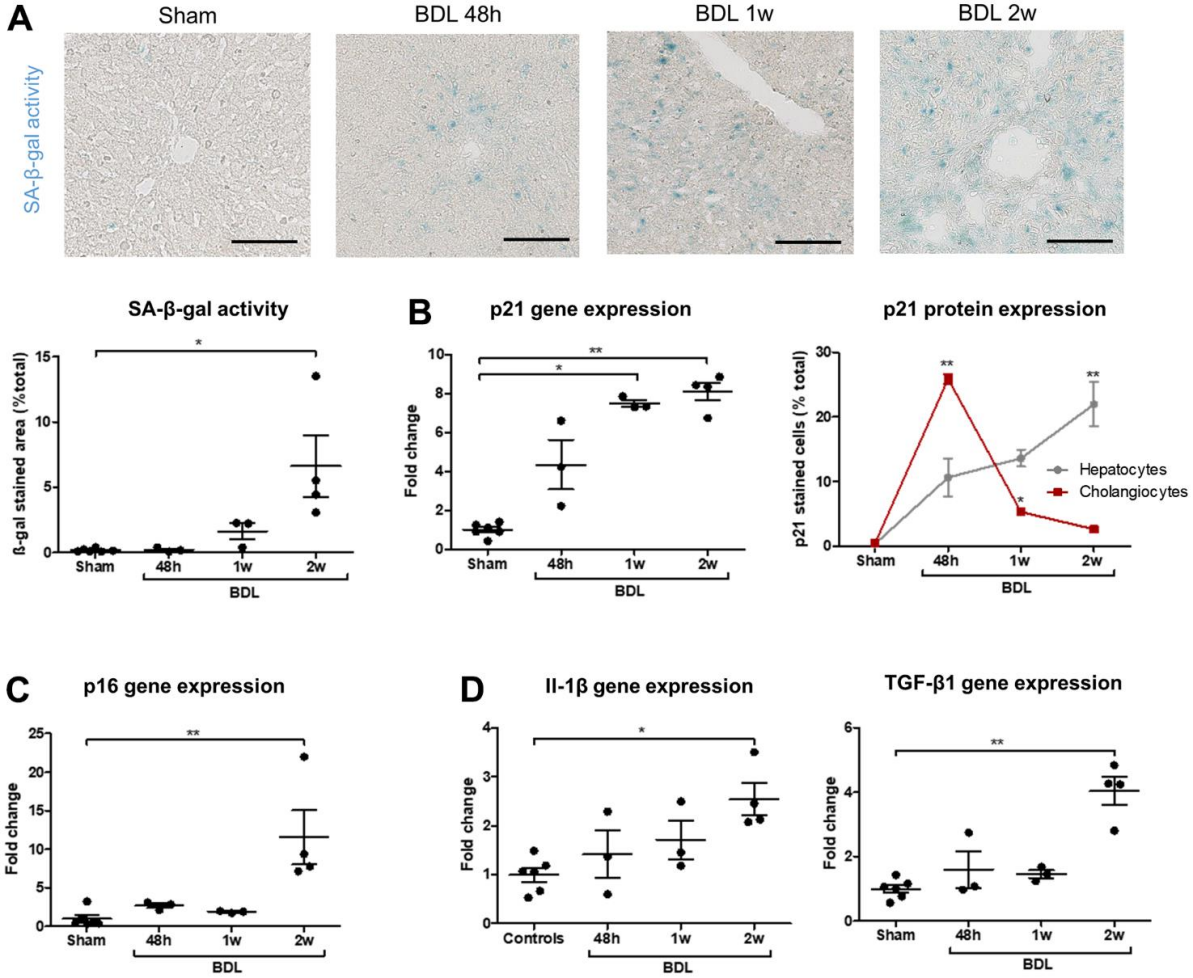
**Senescence progressively develops in BDL rats and appears in cholangiocytes before hepatocytes.** (**A**) SA-β-gal activity increases after the surgery. (**B**) p21 gene expression progressively increase in BDL livers as compared to controls. Senescence (p21-positive cell percentage) is maximal in cholangiocytes 48 hours post-BDL, while hepatocytes senescence increases progressively to become significant only two weeks after the surgery. The percentages of p21-positive cells at different timepoints are compared to the correspondent cellular type of control rats. (**C**) p16 gene expression increases in diseased rats two weeks post-surgery. (**D**) Gene expression of SASP markers Il-1β and TGF-β1 increase in BDL livers as compared to controls. BDL: bile duct ligation; SA-β-gal: senescence-associated β-galactosidase; SASP: senescence-associated secretory phenotype; 48h – 1w – 2w : rats sacrificed 48 hours (n=3) – 1 week (n=3) – 2 weeks (n=4) after BDL surgery. Data is presented as mean ± SEM; *p≤0.05; **p<0.01. Scale bars = 100μm.

Bile duct ligation (BDL) was performed in two-months-old rats to generate a model of biliary senescence in which we could test senotherapeutics. The operated rats developed progressive cholestasis, biliary proliferation, loss of hepatocytes mass and liver fibrosis as expected (Supplementary Figure 6). Senescence progression was confirmed in the animal model as demonstrated by an increased SA-β-gal activity, p21 protein and gene expression, p16 gene expression and SASP-related genes expression (Figure 4). Biliary proliferation and liver fibrosis significantly correlated with senescence development (Supplementary Figure 6A, 6B). As expected, the earliest marker of senescence was p21 as it was significantly increased in whole liver homogenates from 1 week after the surgery (Figure 4B). The percentage of senescent p21-positive cholangiocytes was already maximal after 48 hours and subsequently decreased, while senescence progressively increased in hepatocytes of the parenchyma (Figure 4B).

Our results confirm that BDL is a robust model of biliary senescence. Studying early post-surgical stages allowed us to demonstrate that senescence (p21-positive cells) developed first in cholangiocytes and subsequently in hepatocytes during biliary injury.

### Human allogenic liver-derived progenitor cells but not dasatinib + quercetin decrease early senescence and liver disease in the BDL model

**Figure 5:**
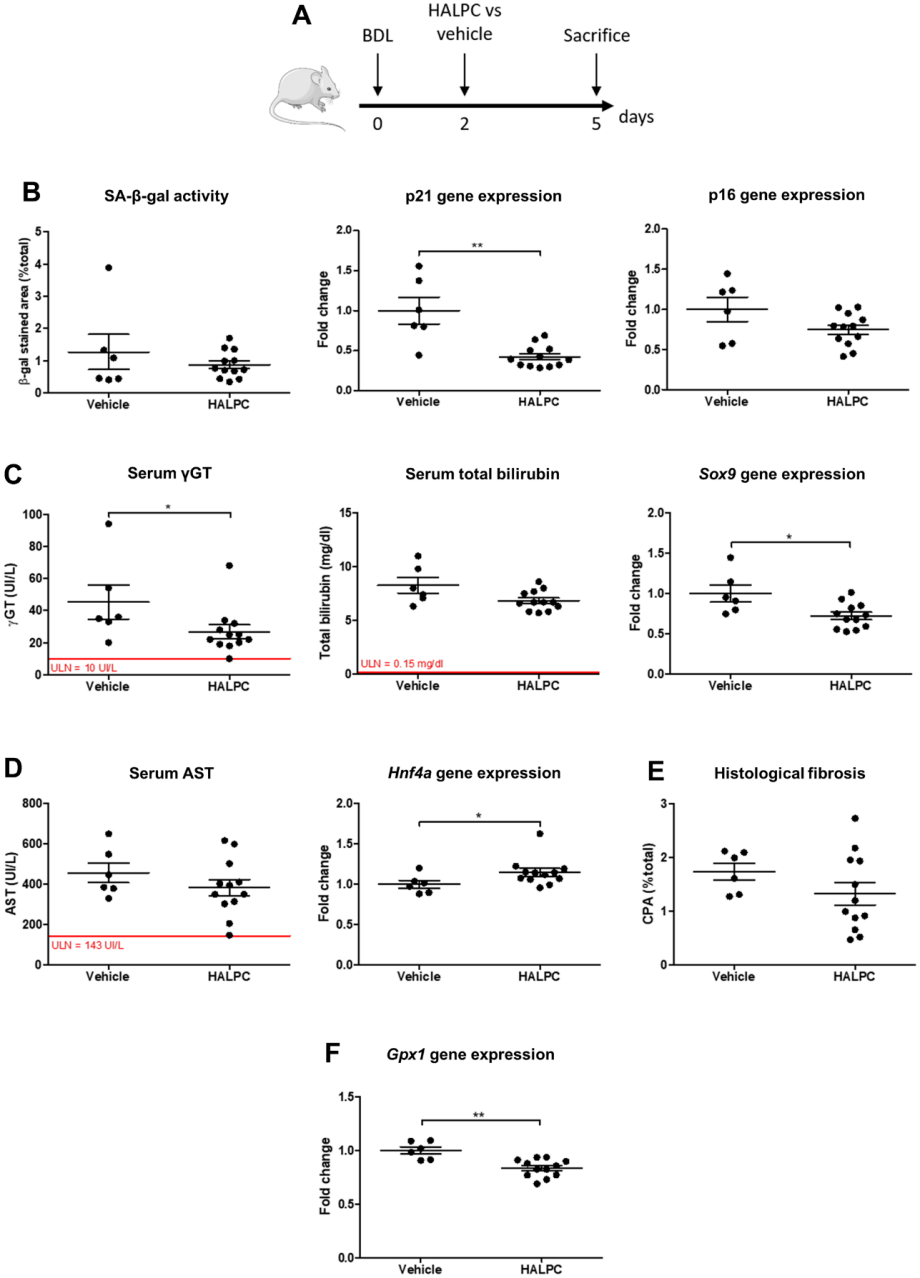
**HALPC administration in BDL rats.** (**A**) Operated rats received HALPC (n=12) through peripheral vein injection 48 hours after BDL and were compared to vehicle-treated controls (n=6). (**B**) HALPC decreased the early marker of senescence p21 gene expression, but no significant effect was observed on later markers (SA-β-gal activity and p16 gene expression). (**C**) HALPC decreased cholangiocytes injury (serum γGT) and biliary proliferation (*Sox9*), but had no significant effect on biochemical cholestasis (serum total bilirubin). (**D**, **E**) HALPC slightly reduced the hepatocytes mass loss (*Hnf4a*) while having no significant effect on serum AST nor on liver histological fibrosis. (**F**) HALPC reduced *Gpx1* expression. BDL: bile duct ligation; HALPC: human allogenic liver-derived progenitor cells; SA-β-gal: senescence-associated β-galactosidase; ULN: upper limit of normal. Data is presented as mean ± SEM; *p≤0.05; **p<0.01.

Once we demonstrated that biliary senescence occurs in the BDL model, we evaluated the efficacy of human allogenic liver-derived progenitor cells (HALPC) or dasatinib (D) + quercetin (Q) in the operated animals. Both therapies were administered 48 hours after the surgery and the animals were compared to vehicle-treated controls (Figure 5A and Supplementary Figure 7A). Since the low dose and the high dose of cells provided comparable results, all the animals injected with HALPC were grouped for further analyses. Intravenous HALPC injection reduced the gene expression of the early marker of senescence p21, while no significant effect was observed regarding SA-β-gal activity nor p16 gene expression (Figure 5B). HALPC improved biliary injury (serum γGT levels) and proliferation (transcription factor SOX-9 – *Sox9* – expression) (Figure 5C). The cells had no effect on the hepatocytes injury (serum AST), but slightly improved the hepatocytes mass loss (hepatocyte nuclear factor 4-alpha - *Hnf4a* – expression) (Figure 5D). No effect was observed on liver histological fibrosis (Figure 5E). The anti-oxidative properties of HALPC were investigated through the gene expression of glutathione peroxidase 1 (*Gpx1*) in liver homogenates and we observed a decrease of *Gpx1* expression in the group that received the cells as compared to the vehicle (Figure 5F). Unexpectedly, the very well described combination of D+Q had no effect on senescence nor liver disease progression as compared to the vehicle and on the opposite appeared to aggravate the hepatocytes mass loss (Supplementary Figure 7).

Our results suggest that HALPC but not D+Q improve early senescence and liver injury in the BDL model of biliary cirrhosis.

## DISCUSSION

Our data provide the first integrative report about premature senescence in pediatric BA and demonstrate that liver senescence and SASP are already present from early stage in BA but continue to progress until liver transplantation, supporting the potential benefit of senotherapeutic interventions following hepatoportoenterostomy. Senescence and SASP were predominant in cholangiocytes as compared to hepatocytes in BA, and senescence progressively developed from cholangiocytes to hepatocytes in the BDL preclinical model of biliary cirrhosis. Furthermore, CCL2 and TGF-β1 – two SASP components that are widely incriminated in the paracrine transmission of senescence – were elevated in our BA livers [9, 29, 30]. Our data corroborate the likelihood of a paracrine transmission of senescence from cholangiocytes to surrounding hepatocytes through the SASP in cholangiopathies, further supporting the use of senotherapies to limit the deleterious effects of the SASP on senescence expansion, tissue remodeling and liver dysfunction [8, 9, 31].

Since we evidenced advanced senescence in BA, the next step was to test senotherapeutics that could be translated to pediatric biliary cirrhosis in the BDL model of biliary cirrhosis. We first demonstrated that the progression of disease and senescence in the liver of operated young rats was in many aspects comparable to BA, before administrating the animals with HALPC or D+Q. We chose to test HALPC – a population of mesenchymal stem cells (MSC) obtained from healthy human liver – in our preclinical model because those cells are already approved for clinical application and because various MSC types already demonstrated to have anti-senescence properties *in vivo* through antioxidant and anti-inflammatory effects [32–35]. However, since HALPC anti-senescence properties remained to be demonstrated, we chose to compare their effect with the very well described combination of the pro-apoptotic drugs D and Q, which was already tested in clinical trials in age-related diseases such as diabetic kidney disease or idiopathic pulmonary fibrosis [36, 37].

HALPC transplantation in BDL young rats improved the early marker of senescence p21, as well as biliary injury and hepatocytes loss, providing encouraging preliminary results regarding the use of senotherapeutics in biliary cirrhosis. HALPC also demonstrated to reduce *Gpx1* expression in our BDL model, possibly due to a cell-mediated reduction of the oxidative stress that could explain a beneficial effect on liver senescence [38]. However, further studies will be needed to truly consider HALPC as senotherapeutics since we observed an improvement of early senescence only with no effect of the cells on the markers of established senescence p16 and SA-β-gal activity nor on liver fibrosis in our short-time experiment. In addition, HALPC are known to secrete numerous bioactive factors with paracrine activity, including anti-inflammatory, anti-fibrotic and regenerative effects [39–43]. The main effects of the cells are known to be mediated by paracrine mechanisms as they displayed pro-regenerative effects in mice livers after partial hepatectomy even in the absence of engraftment and proliferation of their own [43]. We hypothesized that the disease improvement observed in BDL rats was related to the reduction of early senescence by the cells, but other HALPC paracrine properties might also have contributed to this improvement. Proof of efficacy regarding therapeutic interventions that act specifically on senescence and thereby improve liver disease will be needed before translating senotherapies to pediatric biliary cirrhosis.

Unexpectedly, the combination of senolytics D+Q had no effect on senescence nor liver disease progression in our model. Senolytics are – by definition – able to selectively or preferentially kill senescent cells [44]. D+Q induce the selective apoptosis of senescent cells and were shown to decrease hepatocytes senescence and liver steatosis in a preclinical model of non-alcoholic fatty liver disease (NAFLD) [11]. However, a more recent publication showed no effect of D+Q on senescence, liver steatosis nor fibrosis in a model of NAFLD-induced hepatocellular carcinoma [45]. D+Q even seemed to display a pro-tumorigenic effect in this model. In line with this observation, the apoptosis of senescent liver sinusoidal endothelial cells induced perivascular liver fibrosis and health deterioration in aged mice [46]. Also, senescent hepatic stellate cells can limit liver fibrosis and promote liver regeneration in preclinical models [47, 48]. Caution should thereby be advised regarding the use of senolytic therapies in liver disease as consequences could differ according to the removed senescent cell type.

In conclusion, BA livers displayed advanced senescence at diagnosis and senescence and SASP continued to progress until liver transplantation. HALPC but not D+Q reduced early senescence and improved liver disease in a preclinical model of biliary cirrhosis, providing encouraging preliminary results. However, the development of therapeutic agents that target selected senescent cell-types through clearly identified mechanisms and thereby improve established senescence and liver disease will be needed before considering to translate senotherapies to pediatric biliary cirrhosis.

## MATERIALS AND METHODS

### Human samples

Patients who underwent hepatoportoenterostomy procedure (n = 5) or liver transplantation (n = 30) for BA were prospectively recruited in the Pediatric Gastroenterology and Hepatology Unit of Cliniques Universitaires Saint-Luc between 2018 and 2022. A liver biopsy or a fragment of the explanted liver was collected for each patient and obtained from the Cliniques Universitaires Saint-Luc Hepatic Biobank. Biochemical data were obtained the day before the procedure. Control livers (n=10) were obtained from the Cliniques Universitaires Saint-Luc biobank when consent for research purposes was given. Cryopreserved material was available for 5/10 controls.

### Animal model and senotherapeutics administration

Biliary cirrhosis was induced in 2-month-old wild-type male Wistar rats by performing the extrahepatic bile duct ligation-resection surgical procedure as previously described [49]. Controls underwent sham procedure (bile duct dissection without resection) at the same age and were sacrificed at the same time points. Cryopreserved HALPC were obtained from the Hepatic Biobank of Cliniques Universitaires Saint-Luc. The cells were originally derived from the liver of a male donor aged 4 months as previously described and cultured on Corning CellBIND flasks (Merck, Kenilworth, NJ, USA) [50]. For our experiments, cells cryopreserved at P4 were thawed and re-suspended in PBS supplemented with heparin 60UI/10^6^ cells. Cells were subsequently injected to the animals at high dose (12.5x10^6^ cells/kg, n=6) and low dose (1.25x10^6^ cells/kg, n=6) through the penile vein 48 hours after BDL procedure. D (5 mg/kg; Merck) and Q (50 mg/kg; Merck) were diluted in 50% PEG400 and administered by oral gavage 48 hours after BDL surgery (n=5). Controls underwent BDL as well and were either injected with the cell vehicle (n=6) or received 50% PEG400 by oral gavage (n=5) at the same timepoints. All animals were sacrificed three days after the treatment.

### Senescence-associated β-galactosidase activity assay

SA-β-gal activity assay was performed on cryopreserved liver tissue as previously described [51]. The reaction was performed at pH 4 and the staining solution was removed after one hour (for human liver) or two hours (for rat liver) of incubation at 37C. Stained sections were washed with PBS before subsequent immunohistochemical staining when indicated.

### Immunohistochemistry – immunofluorescence

Five μm formalin-fixed paraffin-embedded (FFPE) liver sections were deparaffinized and rehydrated in xylene and graded alcohol series. Antigen retrieval was performed with Tris-EDTA buffer (Merck) or with citrate buffer (Dako Target Retrieval Solution, Agilent, Santa Clara, CA, USA) (Supplementary Table 2). Non-specific immuno-staining was prevented by 1 hour incubation in PBS containing 5% Bovine Serum Albumin (Merck) ± 5% Normal Goat Serum (Thermo Fisher Scientific, Waltham, MA, USA). Thereafter, sections were incubated with primary antibody for 1 hour at 37C (Supplementary Table 2). After washing, sections were incubated with species-specific secondary antibodies (Dako EnVision+ System HRP, Agilent). Immunohistochemical detection was performed with liquid 3,3′-diaminobenzidine (DAB) chromogen and substrate buffer (Dako, Agilent), and was followed by Mayer’s hematoxylin counterstaining. For immunofluorescence staining, tyramide-fluorophore amplification was performed sequentially with different fluorochromes (Alexa-488, 555, 594 or 647, Atto-425, Thermo Fisher Scientific or Invitrogen, Waltham, MA, USA) for each antibody as previously described [52]. Sections were mounted with DAPI containing media or counterstained with Hoechst (Merck) and mounted with Dako fluorescence mounting medium (Dako).

### Imaging and quantification of staining in whole tissue sections

Quantification of SA-β-gal activity staining and immunostaining was assessed as previously described [51]. Briefly, stained slides were digitalized using a SCN400 slide scanner for SA-β-gal activity and immunohistochemistry (Leica Biosystems, Wetzlar, Germany) or Axio Scan.Z1 scanner for immunofluorescence (Zeiss, Oberkochen, Germany) (x20 magnification). Scanned slides were then analyzed using the image analysis tool Author version 2017.2 (Visiopharm, Hørsholm, Denmark) for computer-assisted quantification of histological staining. Results were expressed as (stained area/total tissue area) x 100 to obtain a percentage of stained area for SA-β-gal activity and immunohistochemistry staining, or as (stained cells/total number of cells) x 100 to obtain a percentage of stained cells for immunofluorescence staining. The parameters of the designed Visiopharm APPs were kept constant for all sections. For telomere dysfunction-induced foci evaluation, high magnification images (x100) with z-stack imaging were obtained through spinning disk confocal microscopy (Zeiss) and manual quantification of DNA damage (γH2AX) and/or telomeres (TRF2) foci was performed.

### Reverse transcription quantitative polymerase chain reaction (RT-qPCR)

Liver homogenates were obtained with the FastPrep-24 Classic Instrument (MP Biomedicals, Irvine, CA, USA) from liver biopsies cryopreserved in Lysing Matrix D tubes (MP Biomedicals) filled with Tripure isolation reagent (Roche, Basel, Switzerland). Total RNA was extracted from liver homogenates using Tripure isolation reagent (Roche), according to the manufacturer’s instructions. Genomic DNA was digested by DNase I (Invitrogen). RNA was retro-transcribed using the high-capacity cDNA reverse transcription kit (Applied Biosystems, Waltham, CA, USA). RT-qPCR was carried out in duplicate using TaqMan universal MasterMix (Applied Biosystems) and pre-designed TaqMan probes obtained from Thermo Fisher Scientific or Integrated DNA Technologies (IDT; Coralville, IA, USA) on a StepOnePlus real-time PCR machine (Applied Biosystems) (Supplementary Table 3). Relative gene expression was determined with the ∆∆Ct method using *TBP* and *PPIA* as housekeeping genes for human experiments and *Gapdh* and *B2m* for rat experiments [53].

### Digital spatial whole transcriptome of BA livers

Digital spatial profiling (DSP) GeoMx slide preparation and analysis were performed by the Utrecht Sequencing Facility (USEQ) team (Utrecht, Netherlands). FFPE samples of BA late (n=2), BA early (n=2) and control (n=3) livers were processed and prepared according to the RNA FFPE Manual Slide Preparation Protocol section of the GeoMx NGS Slide Preparation User Manual (MAN-10115-05) by NanoString Technologies (Seattle, WA, USA). Briefly, FFPE slides were baked at 60C for 30 minutes immediately before deparaffinization and rehydration. The slides were then incubated in 100C Tris-EDTA for 15 min to achieve target retrieval. RNA targets were exposed through incubation with Proteinase K at 37C for 15min and a post-fixation step was performed to preserve the samples. The slides were hybridized overnight with GeoMx Human Whole Transcriptome Atlas Human RNA probe mix for Illumina Systems (NanoString Technologies). Stringent washes were performed to remove off-target probes and samples were blocked with Buffer W (NanoString Technologies). Prepared slides were then stained for one hour with immunofluorescent antibodies to allow the identification of tissue morphology and the detection of specific cell types during the ROI selection step. The morphological markers used were pan-cytokeratin (2 μg/ml; NBP2-33200AF532, Novus Biologicals, Centennial, CO, USA), alpha smooth muscle actin (1.25 μg/ml; ab202368, Abcam, Cambridge, UK) and Syto13 (S7575, Thermo Fisher Scientific). Slides were then washed and loaded into the GeoMx DSP machine for scanning (x20 magnification) and ROI selection. Selection of 4-5 ROI for hepatocytes and 4-5 ROI for cholangiocytes was manually performed in each sample. Only the cell type of interest was digitally selected in each ROI thanks to the pan-cytokeratin immunofluorescent staining (high intensity staining for cholangiocytes and low intensity staining for hepatocytes). ROI were validated only if containing at least 100 targeted cells in order to allow sufficient signal for subsequent detection. GeoMx Human Whole Transcriptome Atlas RNA assay contains *in situ* hybridization probes conjugated to unique DNA oligonucleotides (DSP barcodes) via a UV-photocleavable linker. After ROI selection, the DSP barcodes were tagged according to their ROI location and then UV-cleaved and collected to be sequenced on an Illumina sequencer (San Diego, CA, USA). Sequenced oligonucleotides were processed and then imported back into the GeoMx DSP analysis software for integration with the ROI selection information and generation of spatially- and cell type-resolved transcriptomic data.

### Analysis of DSP whole transcriptome data

Two low-performing ROI segments with less than 10% of the genes detected were removed from the analysis (genes were considered as detected when their counts were higher than the LOQ value, defined as the negative probes geometric means + 2 standard deviations). Genes lowly detected across the dataset (in less than 10% of the segments) were removed, leaving 9146 genes for the differential expression analyses. Raw counts were normalized by the Q3 normalization method. Differential expression analyses were performed with DESeq2 Bioconductor package v1.32.0 [54]. Gene set enrichment analyses were done using clusterProfiler v4.0.5 [55].

### Statistical analysis

Statistical analysis was conducted using Graphpad Prism 5.0 (GraphPad Software, La Jolla, CA, USA). Continuous variables were presented as mean ± standard error of the mean (SEM) or median (range) and categorical variables as numbers and percentages. The non-parametric Mann-Whitney U test was used to compare continuous variables between subgroups. When more than two groups were included in the analysis, a non-parametric Kruskal-Wallis One-Way ANOVA with Dunn’s post-hoc test was performed. A two-tailed p-value ≤ 0.05 was considered to indicate statistical significance for all analyses.

## Supplementary Material

Supplementary Figures

Supplementary Tables
