# Supplementary material for: Senescence and senotherapies in biliary atresia and biliary cirrhosis
Source: Aging (Albany NY). 2023 May 18;15(11):4576–99. doi: 10.18632/aging.204700 (PMC10292904; doi:10.18632/aging.204700)
Supplement: Supplementary Figures [file aging-15-204700-s001.pdf]

SUPPLEMENTARY FIGURES

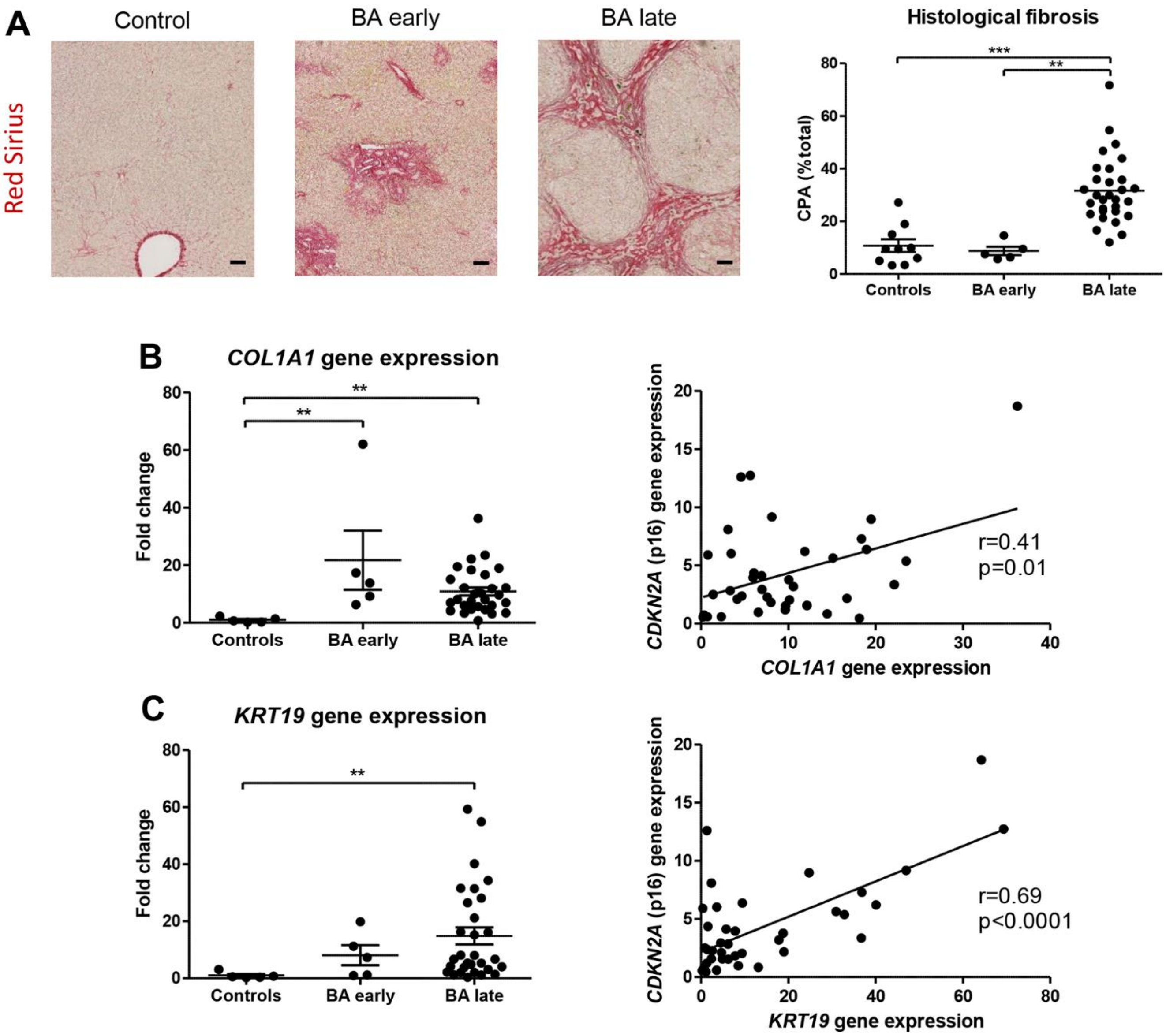

Supplementary Figure 1. Fibrosis and bile ducts mass in BA livers versus controls. (**A**) Histological fibrosis increases in late BA patients compared to controls and to early stage BA; (**B**) Myofibroblasts activation (*COL1A1* expression) increases in BA patients compared to controls and weakly correlates with senescence development; (**C**) Bile ducts mass (*KRT19* expression) increases in BA patients compared to controls and correlates with senescence development. BA: biliary atresia; CPA: collagen proportionate area. Data is presented as mean ± SEM; **p<0.01; ***p<0.001. Scale bars = 100 µm.

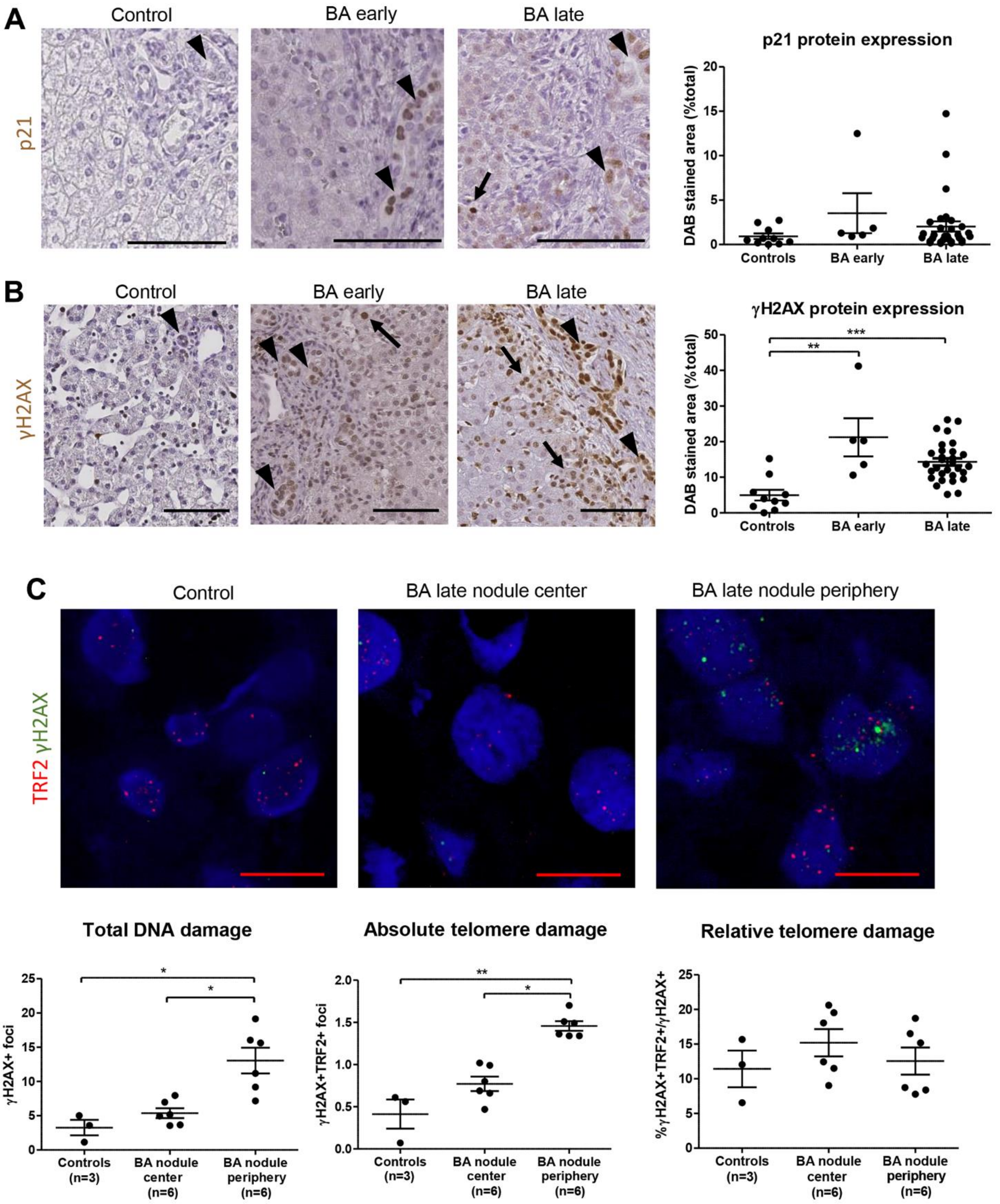

Supplementary Figure 2. DNA and telomere damages increase in BA. (**A**) BA livers do not display an increased p21 protein expression. (**B**) γH2AX protein expression increases in BA and predominates in cholangiocytes (arrowheads) and perinodular hepatocytes (arrows). (**C**) Late stage BA livers display increased DNA damage foci (γH2AX staining) and telomere damage foci (co-localization γH2AX-TRF2) in the periphery of the BA nodules (n=6) as compared to the center of the nodules (n=6) and to controls (n=3). Relative telomere damage (colocalization of γH2AX and TRF2 reported to total DNA damage) do not increase in late BA livers. BA: biliary atresia; DAB: 3,3'-diaminobenzidine. Data is presented as mean ± SEM; *p≤0.05, **p<0.01, ***p<0.001. Black scale bars = 100 µm; red scale bars = 10 µm.

Cholangiocytes: BA early vs controls

gene	log2FC	padj
UBD	3.81	5.69e-77
SLC3A1	-3.02	3.33e-61
SCGB3A1	-2.87	5.51e-46
TFF3	-3.11	5.66e-46
IL32	3.44	3.17e-45
KCNJ15	-2.60	1.37e-38
PRMT8	-1.99	2.64e-37
MUC1	-2.75	2.95e-37
ACE2	-2.37	5.30e-36
MPP6	-1.91	6.69e-36

451 DE genes (padj < 0.05 and |log2FC| > 1)

Hepatocytes: BA early vs controls

gene	log2FC	padj
UBD	3.04	1.96e-111
PPP2R1B	2.12	4.05e-94
IL32	3.27	6.40e-87
TGFBI	2.79	5.16e-77
IGF2	6.52	2.61e-73
GPC3	4.10	6.90e-73
HSD11B1	-2.53	3.80e-72
ACSL4	3.82	4.76e-63
SPP2	2.34	6.60e-60
CFB	2.33	1.89e-56

347 DE genes (padj < 0.05 and |log2FC| > 1)

Cholangiocytes: BA late vs controls

gene	log2FC	padj
SLC3A1	-3.00	1.11e-58
UBD	3.34	1.17e-58
MCAM	3.03	5.08e-58
BACE2	2.53	5.36e-57
MPP6	-2.49	8.47e-55
TFF3	-3.38	8.36e-53
KCNJ15	-3.15	1.17e-50
CXCL6	3.99	4.58e-48
SCGB3A1	-2.79	1.47e-43
ACE2	-2.64	8.97e-42

518 DE genes (padj < 0.05 and |log2FC| > 1)

Hepatocytes: BA late vs controls

gene	log2FC	padj
LEPR	3.11	9.00e-100
MFSD2A	2.12	4.25e-59
CFB	2.24	6.25e-49
ABCB4	2.34	1.01e-46
A1BG	2.05	2.07e-46
TNFSF14	1.81	1.06e-45
RNASE4	1.42	8.10e-40
PPP2R1B	1.43	1.13e-39
C8G	1.37	1.58e-39
ITIH3	2.74	8.55e-39

228 DE genes (padj < 0.05 and |log2FC| > 1)

Cholangiocytes: BA late vs BA early

gene	log2FC	padj
TNC	2.35	2.03e-70
BACE2	1.61	2.03e-70
KRT23	2.35	2.37e-29
MCAM	1.25	9.20e-29
KRT81	1.87	9.20e-29
CCND2	1.40	1.88e-24
PPP1R12B	1.11	1.07e-22
CLU	-2.09	6.92e-22
GDA	1.46	2.36e-20
IGF2	-3.43	1.13e-18

90 DE genes (padj < 0.05 and |log2FC| > 1)

Hepatocytes: BA late vs BA early

gene	log2FC	padj
MFSD2A	2.89	1.29e-101
HSD11B1	2.41	3.97e-62
SLC1A1	1.72	1.41e-57
GPC3	-3.56	3.35e-53
UBD	-2.01	1.27e-50
ZNF771	-1.25	1.27e-43
NNMT	4.39	2.22e-41
TNFRSF10C	-1.19	3.58e-37
PLIN2	2.64	7.47e-34
ADAM15	-1.00	6.78e-30

145 DE genes (padj < 0.05 and |log2FC| > 1)

Supplementary Figure 3. Differential expression analysis between subgroups in BA spatial WTA dataset. Total number of differentially expressed genes (padj < 0.05 and |log2FC| > 1) is mentioned for each subgroup comparison. Detailed data are listed for the top 10 main significantly modified genes in each subgroup comparison. BA: biliary atresia; DE: differentially expressed; FC: fold change; padj: adjusted p-value; WTA: whole transcriptome analysis.

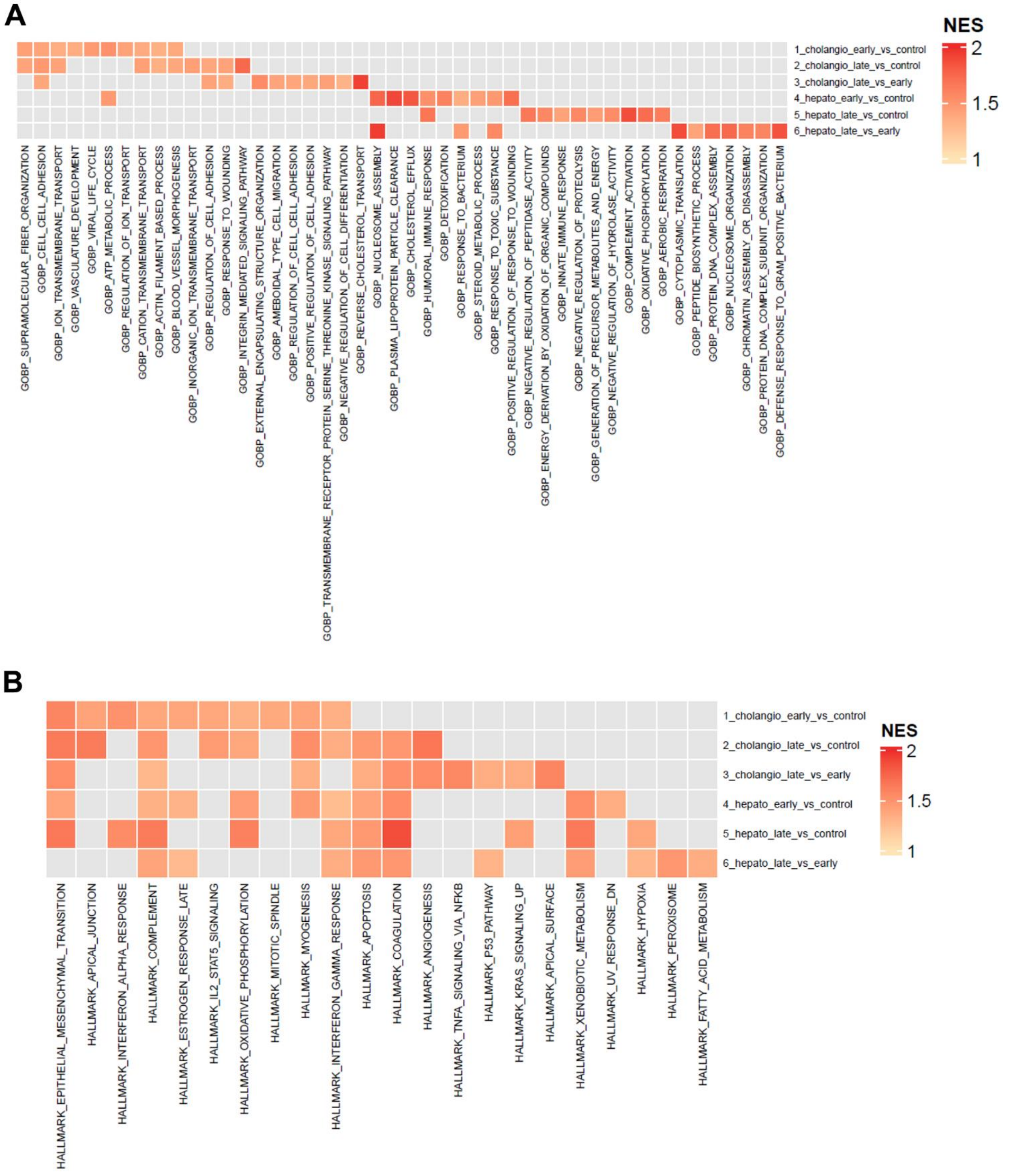

Supplementary Figure 4. GSEA between subgroups in BA spatial WTA dataset. The ten most significantly enriched GO terms (**A**) or hallmark gene lists (**B**) are represented for each subgroup comparison. BA: biliary atresia; GO: gene ontology; GSEA: gene set enrichment analysis; NES: normalized enrichment score; WTA: whole transcriptome analysis.

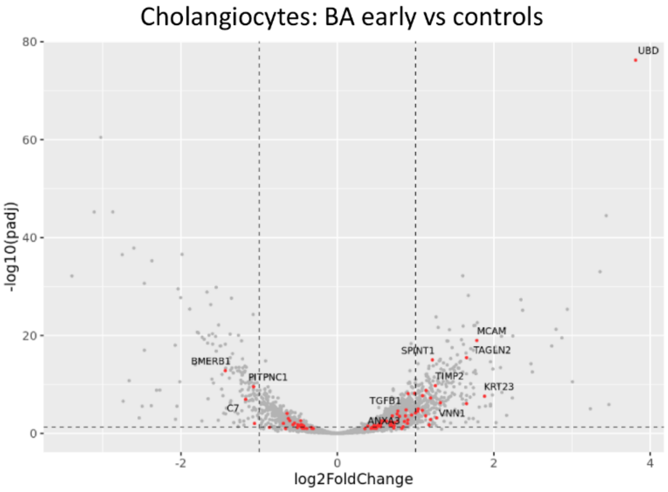

Gene	Log2FC	padj
UBD	3,81	5,69E-77
MCAM	1,78	1,01E-19
TAGLN2	1,65	3,23E-16
SPINT1	1,21	9,26E-16
BMERB1	-1,43	1,46E-13
TIMP2	1,25	1,74E-10
PITPNC1	-1,07	2,82E-10
RHOC	1,13	1,68E-9
S100A11	0,99	6,26E-9
TAX1BP3	0,90	7,20E-9
HSPG2	1,09	1,97E-8
KRT23	1,88	2,49E-8
DTNA	1,19	5,65E-8
C7	-1,17	1,08E-7
C1S	1,32	5,37E-7
VNN1	1,65	8,22E-7
PLK2	1,03	1,02E-5
TGFB1	0,88	1,52E-5
LAPTM5	1,09	1,90E-5

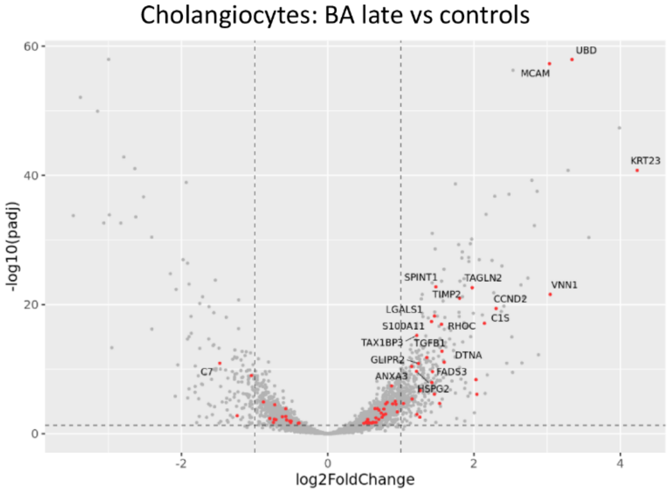

Gene	Log2FC	padj
UBD	3,34	1,17E-58
MCAM	3,03	5,08E-58
KRT23	4,23	1,69E-41
SPINT1	1,48	1,82E-23
TAGLN2	1,98	2,47E-23
VNN1	3,04	2,66E-22
TIMP2	1,80	1,04E-21
CCND2	2,30	4,11E-20
LGALS1	1,46	5,96E-19
S100A11	1,42	4,19E-18
C1S	2,14	8,06E-18
RHOC	1,55	1,13E-17
TAX1BP3	1,22	6,08E-16
DTNA	1,56	1,67E-13
TGFB1	1,35	1,6E-12
FADS3	1,59	8,38E-12
C7	-1,48	1,18E-11
GLIPR2	1,24	1,31E-11
ANXA3	1,15	3,82E-11
HSPG2	1,21	2,17E-10

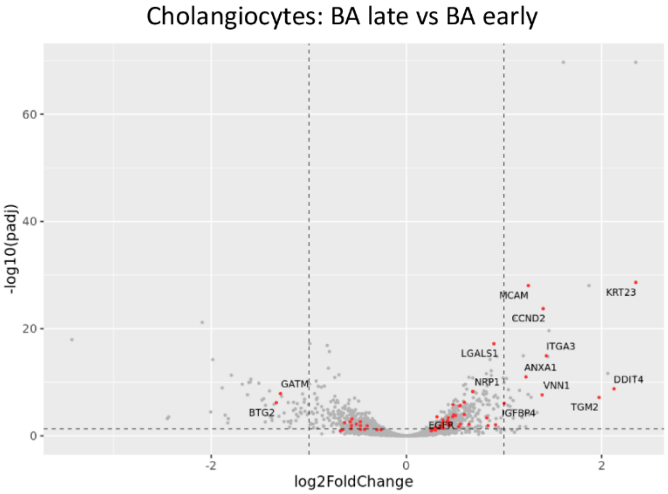

Gene	Log2FC	padj
KRT23	2,35	2,36E-29
MCAM	1,25	9,20E-29
CCND2	1,40	1,88E-24
LGALS1	0,90	6,57E-18
ITGA3	1,43	1,16E-15
ANXA1	1,23	1,02E-11
DDIT4	2,13	1,68E-9
NRP1	0,68	5,27E-9
GATM	-1,29	1,30E-8
VNN1	1,39	2,26E-8
TGM2	1,97	6,76E-8
FADS3	0,59	5,60E-7
BTG2	-1,33	6,41E-7
IGFBP4	1,00	8,44E-7
GLIPR2	0,48	1,49E-6
TIMP2	0,55	2,12E-6
RHBDF2	0,55	2,67E-6
PHLDA3	0,59	1,01E-4
CAPN2	0,48	1,17E-4
EGFR	0,50	1,63E-4

Supplementary Figure 5. Senescence enriched genes in BA spatial WTA dataset. Significant enrichment of a published gene list in senescent HepG2 cells (Aravinthan et al. 2014) was observed in all cholangiocytes subgroups (BA early vs controls; BA late vs controls; BA late vs BA early), indicating a progression of cholangiocytes senescence associated with disease stage in BA. The leading edge genes of the GSEA are highlighted in red in the volcano plots of each subgroup comparison. Detailed data are listed for the top 20 leading edge genes in each subgroup comparison. BA: biliary atresia; FC: fold change; GSEA: gene set enrichment analysis; padj: adjusted p-value; WTA: whole transcriptome analysis.

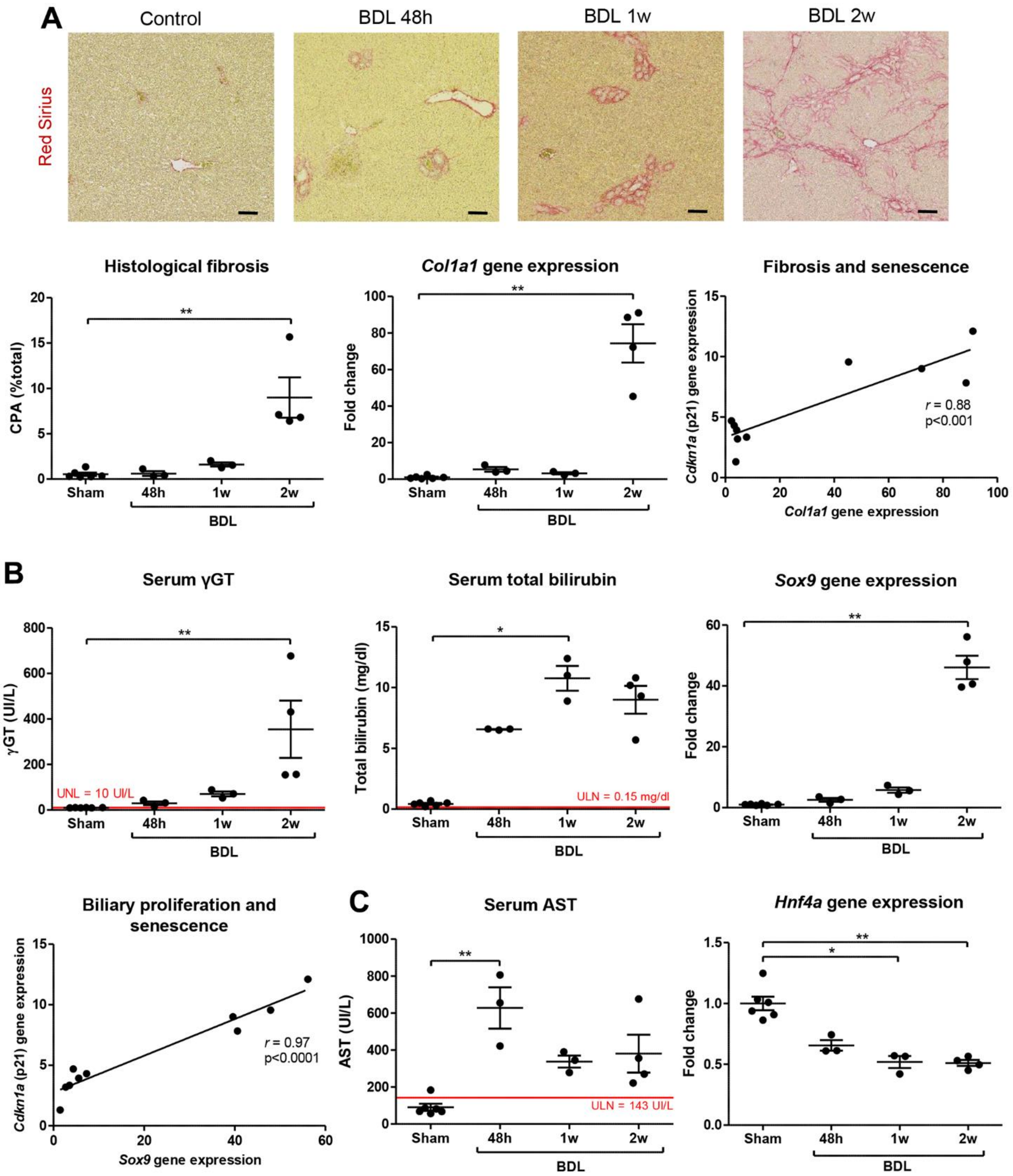

Supplementary Figure 6. Liver disease is correlated to senescence progression in BDL. (**A**) Liver fibrosis increases post-BDL and correlates with senescence progression. (**B**) Biliary damage (serum γGT) and proliferation (*Sox9*) increase in BDL rats and biliary proliferation correlates with senescence progression. (**C**) Serum AST maximal increase occurs 48 hours post-BDL and is followed by a loss of hepatocytes mass (*Hnf4a*). BDL: bile duct ligation; CPA: collagen proportionate area; 48h – 1w – 2w: rats sacrificed 48 hours (n=3) – 1 week (n=3) – 2 weeks (n=4) after BDL surgery. Data is presented as mean ± SEM; *p≤0.05; **p<0.01. Scale bars = 100µm.

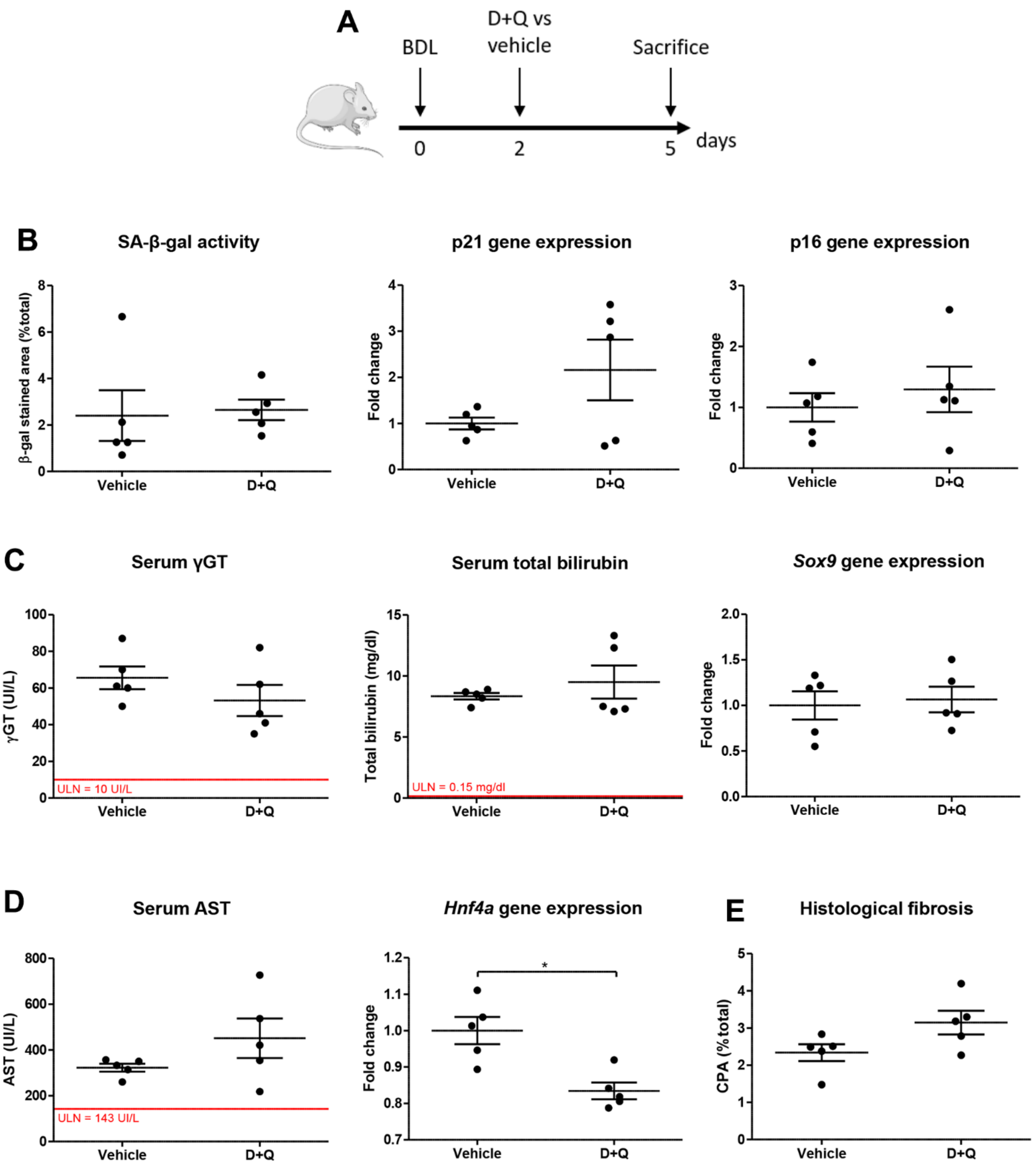

Supplementary Figure 7. D+Q administration in BDL rats. (**A**) Operated rats received D (5 mg/kg) + Q (50 mg/kg) by oral gavage (n=5) 48 hours after BDL and were compared to controls that received the vehicle (50% PEG400; n=5). (**B**) D+Q had no effect on liver senescence. (**C**) D+Q had no effect on biliary injury (serum γGT) and proliferation (*Sox9*) nor on biochemical cholestasis (serum total bilirubin). (**D**, **E**) D+Q worsened the hepatocytes mass loss (*Hnf4a*) and had no significant effect on hepatocytes injury (serum AST) nor on liver histological fibrosis. BDL: bile duct ligation; D: dasatinib: Q: quercetin; SA-β-gal: senescence-associated β-galactosidase; ULN: upper limit of normal. Data is presented as mean ± SEM; *p≤0.05.
