# Supplementary material for: Senescence and senotherapies in biliary atresia and biliary cirrhosis
Source: Aging (Albany NY). 2023 May 18;15(11):4576–99. doi: 10.18632/aging.204700 (PMC10292904; doi:10.18632/aging.204700)
Supplement: Supplementary Tables [file aging-15-204700-s002.pdf]

SUPPLEMENTARY TABLES

Supplementary Table 1. Description of the study population.

	Controls (n=10)	BA early (n=5)	BA late (n=30)
Median age	9.5 y (3 d - 41 y)	7 w (17 d - 2 m)	12 m (6 m - 15 y)
Gender (M/F)	5 (50%) / 5	3 (60%) / 2	15 (50%) / 15
AST (UI/L) (< 80)	NA	232 ± 78	228 ± 19
ALT (UI/L) (< 35)	NA	155 ± 57	123 ± 11
γGT (UI/L) (< 40)	NA	455 ± 78	221 ± 30
Total bilirubin (mg/dL) (< 1.2)	NA	9.1 ± 1.7	17.5 ± 1.7
INR (0.8-1.2)	NA	1 ± 0.04	1.5 ± 0.1
Histological fibrosis (Metavir score):			
F0	10 (100%)	1 (20%)	0
F1	0	0	0
F2	0	1 (20%)	0
F3	0	1 (20%)	0
F4	0	2 (40%)	30 (100%)

In the first column, standard values are indicated for all biochemical parameters. Continuous data is presented as median (range) or mean ± SEM. D, days; m, months; NA, not available; y, years.

Supplementary Table 2. Primary antibodies.

Primary antibody (target)	Company	Cat. no	Species	Ag retrieval	Dilution
p21 WAF1/Cip1 (H)	Agilent	M7202	M mAb	T 1 hour 98C	IHC 1/400
p21 WAF1/Cip1 (R)	Agilent	M7202	M mAb	T 900W 4 min - 90W 15 min - 900W 1.5 min<br>T Retriever 2100	IHC 1/50<br>IF 1/50
p16 INK4a (H)	Abcam	ab108349	Rb mAb	T 1 hour 98C	IHC 1/1000
gamma H2A.X (Ser139) (H)	Abcam	ab81299	Rb mAb	C 35 min 98C<br>C Retriever 2100	IHC 1/500<br>IF 1/500
gamma H2A.X (Ser139) (H)	Merck	05-636	M mAb	C 5 min 450W	IF 1/1000
TRF2 (H)	Novus	NB110-57130	Rb pAb	C 5 min 450W	IF 1/2000
HNF4α (H-R)	R&D System	PP-H1415	M mAb	C Retriever 2100	IF 1/400
CK19 (H)	Dako	M0888	M mAb	C 35 min 98C<br>C Retriever 2100	IHC 1/100<br>IF 1/100
CK19 (R)	Abcam	ab220193	M mAb	C Retriever 2100	IF 1/200

Ag, antigen; C, citrate; H, human; IF, immunofluorescence; IHC, immunohistochemistry; M, Mouse; mAb, monoclonal antibody; pAb, polyclonal antibody; R, rat; Rb, rabbit; T, Tris-EDTA.

Supplementary Table 3. Pre-designed TaqMan probes used for reverse transcription quantitative polymerase chain reaction.

Gene of interest (specie)	Company	Reference
CDKN1A (H)	Thermo Fisher Scientific	Hs00355782_m1
CDKN2A (H)	Thermo Fisher Scientific	Hs00923894_m1
CXCL8 (H)	IDT	Hs.PT.58.38869678.g
TGFB1 (H)	IDT	Hs.PT.58.39813975
COL1A1 (H)	Thermo Fisher Scientific	Hs00164004_m1
KRT19 (H)	Thermo Fisher Scientific	Hs00761767_s1
HNF4A (H)	Thermo Fisher Scientific	Hs00230853_m1
TBP (H)	Thermo Fisher Scientific	Hs99999910_m1
PPIA (H)	Thermo Fisher Scientific	Hs99999904_m1
Cdkn1a (R)	IDT	Rn.PT.58.12154239
Cdkn2a (R)	Thermo Fisher Scientific	Rn00580664_m1
Il1b (R)	IDT	Rn.PT.58.38028824
Tgfb1 (R)	IDT	Rn.PT.58.6690138
Col1a1 (R)	IDT	Rn.PT.58.7562513
Sox9 (R)	IDT	Rn.PT.58.29440750
Hnf4a (R)	IDT	Rn.PT.58.34229621.g
Gpx1 (R)	IDT	Rn.PT.58.7204268.g
Gapdh (R)	Thermo Fisher Scientific	Rn01775763_g1
B2m (R)	Thermo Fisher Scientific	Rn00560865_m1
